# Supplementary figures and images for: Chemical range recognized by the ligand-binding domain in a representative amino acid-sensing taste receptor, T1r2a/T1r3, from medaka fish
Source: PLoS One. 2024 Mar 22;19(3):e0300981. doi: 10.1371/journal.pone.0300981 (PMC10959364; doi:10.1371/journal.pone.0300981)

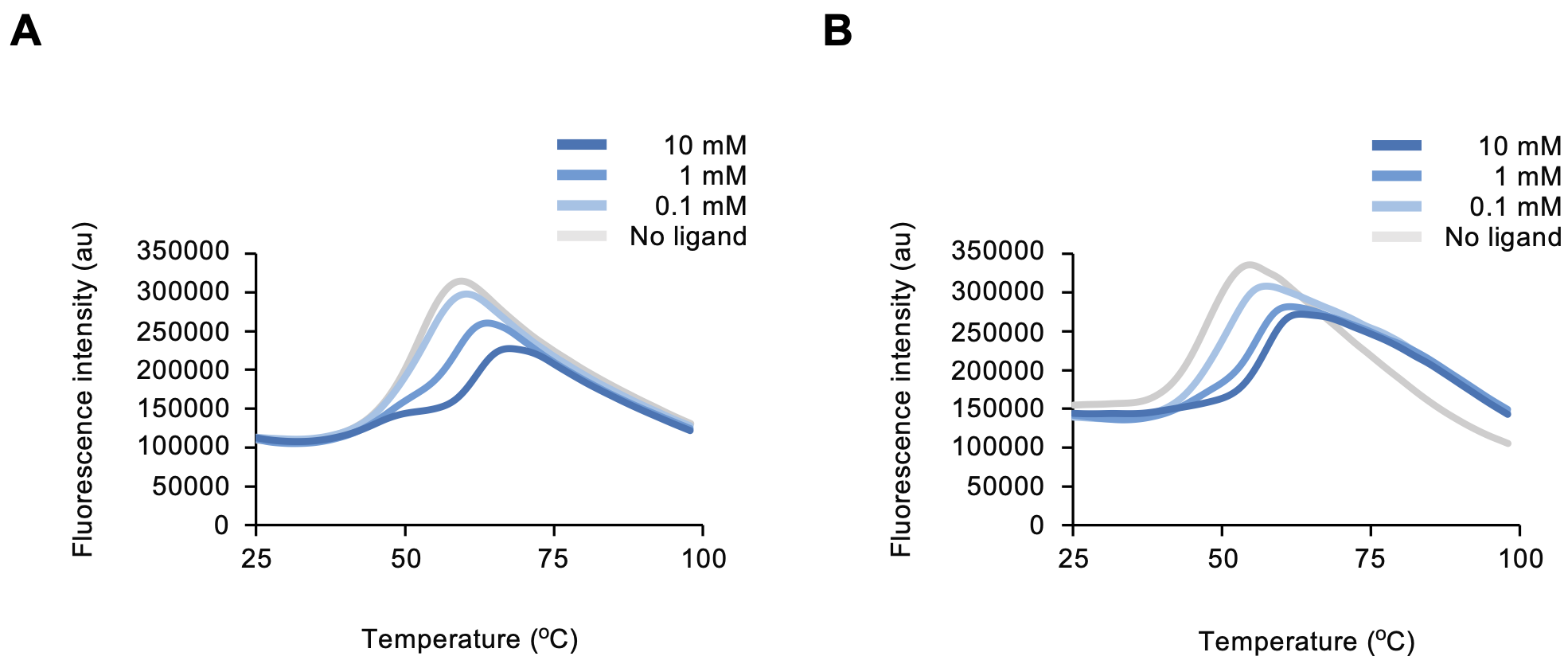

Supplement: S1 Fig — (A) Representative melting curves in the assay buffer. The Tm values derived from four technical replicated measurements represent no ligand, 53.0 ± 0.16°C; 0.1 mM l-alanine added, 53.4 ± 0.12°C (ΔTm, 0.4°C); 1 mM l-alanine added, 58.2 ± 0.29°C (ΔTm, 5.2°C); 10 mM l-alanine added; 62.0 ± 0.02°C (ΔTm, 9.0°C). (B) Representative melting curves in the assay buffer containing 10% DMSO. The Tm values derived from four technical replicated measurements represent no ligand, 47.3 ± 0.23°C 0.1 mM l-alanine added, 50.9 ± 0.50°C (ΔTm, 3.6°C); 1 mM l-alanine added, 55.9 ± 0.16°C (ΔTm, 8.6°C); 10 mM l-alanine added; 57.5 ± 0.02°C (ΔTm, 10.2°C). (TIF) [file pone.0300981.s001.tif]

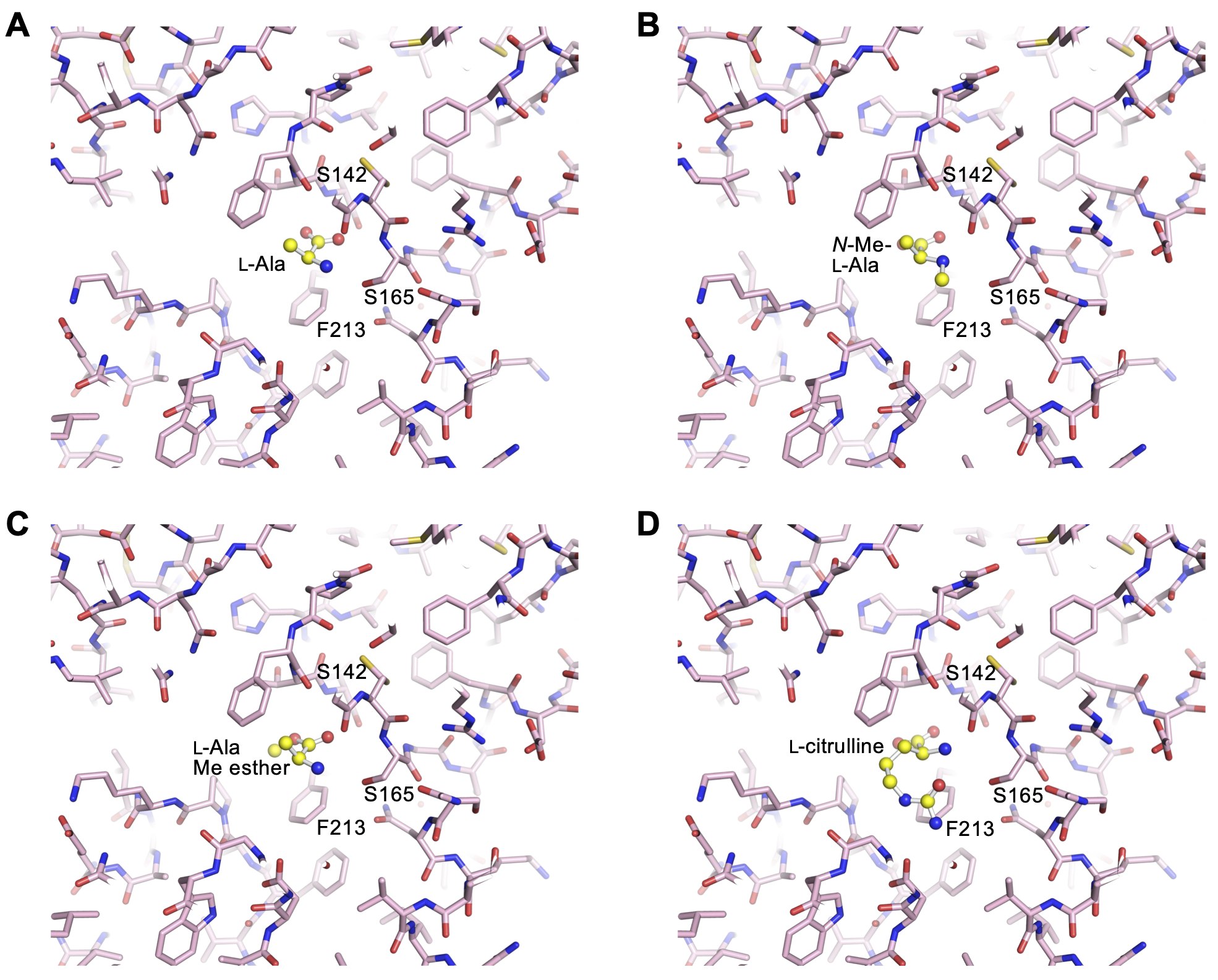

Supplement: S2 Fig — (A) A close-up view of the ligand-binding site in T1r2a in the crystal structure of the l-alanine-bound form (PDB ID: 5X2N). (B–D) Representative docking poses of N-methyl-l-alanine (N-Me-l-Ala; ZINC 901468; B), l-alanine methyl ester (l-Ala Me ester; ZINC 34702232; C), and l-citrulline (ZINC 1532614; D) in the ligand-binding site of T1r2a. In panels B–D, the docking simulations were performed using SwissDock (Grosdidier et al. Nucleic Acids Res. 39, W270, 2011), using the coordinates of T1r2aLBD (PDB ID: 5X2N, chain A) without ligands as a target. (TIF) [file pone.0300981.s002.tif]
